# TE = 32 ms vs TE = 100 ms echo‐time ^1^H‐magnetic resonance spectroscopy in prostate cancer: Tumor metabolite depiction and absolute concentrations in tumors and adjacent tissues

**DOI:** 10.1002/jmri.24875

**Published:** 2015-08-10

**Authors:** Meer Basharat, Geoffrey S. Payne, Veronica A. Morgan, Chris Parker, David Dearnaley, Nandita M. deSouza

**Affiliations:** ^1^ CRUK and EPSRC Cancer Imaging Centre, Institute of Cancer Research and Royal Marsden NHS Foundation TrustSuttonSurreyUK; ^2^ Academic Urology Unit, Institute of Cancer Research and Royal Marsden NHS Foundation TrustSuttonSurreyUK

**Keywords:** magnetic resonance spectroscopy, prostate cancer, citrate, spermine

## Abstract

**Purpose:**

To compare the depiction of metabolite signals in short and long echo time (TE) prostate cancer spectra at 3T, and to quantify their concentrations in tumors of different stage and grade, and tissues adjacent to tumor.

**Materials and Methods:**

First, single‐voxel magnetic resonance imaging (MRI) spectra were acquired from voxels consisting entirely of tumor, as defined on *T*_2_‐weighted and diffusion‐weighted (DW)‐MRI and from a biopsy‐positive octant, at TEs of 32 msec and 100 msec in 26 prostate cancer patients. Then, in a separate cohort of 26 patients, single‐voxel TE = 32 msec MR spectroscopy (MRS) was performed over a partial‐tumor region and a matching, contralateral normal‐appearing region, defined similarly. Metabolite depiction was compared between TEs using Cramér‐Rao lower bounds (CRLB), and absolute metabolite concentrations were calculated from TE = 32 msec spectra referenced to unsuppressed water spectra.

**Results:**

Citrate and spermine resonances in tumor were better depicted (had significantly lower CRLB) at TE = 32 msec, while the choline resonance was better depicted at TE = 100 msec. Citrate and spermine concentrations were significantly lower in patients of more advanced stage, significantly lower in Gleason grade 3+4 than 3+3 tumors, and significantly lower than expected from the tumor fraction in partial‐tumor voxels (by 14 mM and 4 mM, respectively, *P* < 0.05).

**Conclusion:**

Citrate and spermine resonances are better depicted at short TE than long TE in tumors. Reduction in these concentrations is related to increasing tumor stage and grade in vivo, while reductions in the normal‐appearing tissues immediately adjacent to tumor likely reflect tumor field effects. J. Magn. Reson. Imaging 2015;42:1086–1093.

Increasingly, magnetic resonance imaging (MRI) is used as the primary imaging modality for detecting and staging prostate cancer. However, while morphological MRI techniques such as *T*_2_‐ and diffusion‐weighted (DW)‐MRI are effective at identifying large prostate tumors in the peripheral zone, they are less effective at detecting small tumors^1^, ^2^ and in differentiating central gland tumors from benign disease. Additionally, the biological aggressiveness of prostate tumors (which equates with histological Gleason grade) is indistinguishable using conventional morphological MRI techniques.^3^, ^4^ Gleason grade is important for patient prognosis^5^ and therefore for planning management, but as it is reliant on tissue biopsies that are subject to random sampling error, it may be incorrectly represented.^6^

Magnetic resonance spectroscopy (MRS) can provide complementary information in the same patient examination by measuring tissue metabolite levels. In ex vivo prostate tumor extracts the concentrations of metabolites from the glandular secretions (citrate and spermine) are reduced compared with normal prostate tissue, while that of the cell membrane precursor choline is increased.^7^ Unfortunately, quantifying metabolite signals from in vivo prostate spectra is challenging due to the inherently small signals present at clinical field strengths. Moreover, owing to the overlap of spectral peaks, the composite metabolite ratio (choline + spermine + creatine)/citrate is usually measured from prostate spectra, using integration. There are only a few instances where absolute concentrations of citrate, creatine, and choline have been measured, using either integration or metabolite fitting.^8^, ^9^, ^10^, ^11^, ^12^

In vivo prostate MRS typically employs echo times (TEs) greater than 130 msec in order to accommodate radiofrequency pulses for lipid suppression into the spectroscopic sequence, and to allow the four *J*‐coupled peaks of the citrate resonance to be in phase (and therefore integrated across). However, all citrate peaks are also fully upright at TEs of ∼30 msec, and fully inverted at TEs of ∼100 msec. In a previous study, citrate, spermine, choline, creatine, and myo‐inositol signals were measurable individually in greater than 75% of the lipid‐free voxels from normal‐appearing prostate tissues using TE = 32 msec,^13^ and the depiction of metabolite resonances was better at TE = 32 msec than at TE = 100 msec due to reduced *T*_2_‐decay and *J*‐evolution at the shorter TE. However, the depiction and concentrations of metabolites in prostate tumor spectra at short TE has not yet been documented. Citrate and spermine concentrations decrease in tumor, so it is important to determine whether their signals can still be depicted in tumor and, if they can, what these concentrations can tell us about the normal‐appearing tissues adjacent to tumor, which may become metabolically abnormal before morphological changes appear.^14^

The aims of this pilot study were to 1) compare the depiction of metabolite resonances from prostate tumor tissues at the shortest TE available, TE = 32 msec, with that at TE = 100 msec; 2) quantify the concentration of metabolites in prostate tumors using TE = 32 msec MRS, establishing any differences with tumor stage or grade; and 3) compare metabolite concentrations in tissues adjacent to morphologically identified cancer with those in matching, contralateral normal‐appearing voxels.

## Subjects and methods

### Subjects

Fifty‐two prostate cancer patients were studied under a protocol approved by our Institutional Research Ethics Committee. All patients had low‐risk tumors being managed by active surveillance, with prostate cancer proven by randomly sampled 10‐core transrectal ultrasound‐guided biopsy performed at least 2 months before imaging. Due to the time constraints of the imaging examination, patients were studied in two cohorts. Cohort 1 consisted of 26 patients who had single‐voxel MRS data acquired at TE = 32 msec and again at TE = 100 msec, in tissues consisting entirely of tumor, as defined on *T*_2_‐weighted and DW‐MRI in biopsy‐positive octants. The age of these patients was 71 ± 5 years (mean ± SD), tumor stages T1 (*n* = 7), T2 (*n* = 11), and T3 (*n* = 8), and Gleason grades 3+3 (*n* = 6), 3+4 (*n* = 10), 4+3 (*n* = 9), and 3+5 (*n* = 1). Cohort 2 consisted of 26 patients who had single‐voxel MRS at TE = 32 msec data acquired from a tumor region and a matching, contralateral region of normal‐appearing prostate (as defined on *T*_2_‐W and DW‐MRI from a biopsy positive or negative octant, respectively). The age of these patients was 69 ± 7 years (mean ± SD), with tumor stages T1 (*n* = 18), T2 (*n* = 4), and T3 (*n* = 4), and Gleason grades 3+3 (*n* = 14), 3+4 (*n* = 9), 4+3 (*n* = 2), and 4+5 (*n* = 1).

### MRI

Patients were examined on a 3.0T Achieva TX scanner (Philips Medical Systems, Best, The Netherlands) using an endorectal coil (Medrad, Pittsburgh, PA) inflated with 60 mL of perfluorocarbon in combination with a phased‐array surface coil. For *T*_2_W‐MRI, a turbo spin‐echo sequence was used to acquire MR images in the transverse plane, using the following parameters: TR/TE = 3643/110 msec; four averages; matrix size, 220 × 184; slice thickness, 2.2 mm; slice separation, 0.1 mm; right–left field of view, 140 mm. DW‐MRI was performed with an echo‐planar sequence with matrix size, 176 × 176; slice thickness, 2.2 mm; slice separation, 0.1 mm; transverse field of view, 100 × 100 mm, b‐values 0, 100, 300, 500, and 800 s/mm^2^. Apparent diffusion coefficient (ADC) value maps were computed using a monoexponential fit. (Although we accept that it is commonly standard practice to exclude b = 0 from ADC calculations in order to reduce perfusion effects, that was not regarded necessary here since ADC values were only used to aid tumor identification.)

### MRS

In all cases, MRS was performed using single‐voxel point‐resolved spectroscopy (PRESS) with automated shimming, using pulses with frequency centered on citrate (2.62 ppm), 90–180° time = 9 msec, and repetition time = 1410 msec, lasting roughly 3.5 minutes per sequence. The average voxel sizes in Cohorts 1 and 2 were 2.4 ± 1.1 cm^3^ and 3.5 ± 0.9 cm^3^, respectively. MR echoes were acquired using 2048 time domain points, bandwidth = 2000 Hz, number of acquisitions = 128, and receiver gain manually set to 0 dB on all scans. Frequency‐selective pulses followed by gradient spoilers were used to null the water signal at the start of PRESS. No lipid‐suppression protocols were used. For TE = 32 msec spectra only, equivalent scans were also performed without any water suppression; these scans were performed using pulses with frequency centered on water (4.7 ppm) in order to provide water signal for concentration referencing.

### Spectral Processing

Spectral data were processed offline using jMRUI v4.0 (www.mrui.uab.es/mrui/). After zero filling, the Hankel–Lanczos singular value decomposition (HLSVD) filter was used to remove residual water above 4.1 ppm and lipid signals below 1.65 ppm. Metabolite quantification was accomplished with quantitation using quantum estimation (QUEST). QUEST was performed with citrate, spermine, choline (to represent total choline from free choline, phosphocholine, and glycerophosphocholine), creatine, and myo‐inositol metabolite models^13^ and singlet peaks at 2.1, 3.7, and 4.1 ppm in order to fit the resonances of other overlapping spin species (lipids, the minor polyamines spermidine and putrescine, glutamate, glutamine, and lactate). The background handling approach *Subtract* was employed with one truncation point in the raw data as advocated previously.^13^ It was not possible to find a valid model to fit citrate at TE = 100 msec.^13^ In every spectrum, the frequency positions and linewidths of the model resonances were independently and automatically adjusted from their original simulation values to fit to the data; frequency shifting was limited to between −0.03 and +0.03 ppm and linewidth broadening was limited to between 0 and 11.1 Hz.

A measure of metabolite depiction was provided by the Cramér‐Rao Lower Bound (CRLB), whose value provides the minimum uncertainty in the metabolite fit, given the data quality and the model. The CRLB is always larger than zero, and any values of CRLB greater than the metabolite amplitude are redundant. Since the distribution of CRLB/amplitude is nonnormal, median CRLB values and interquartile ranges are reported using Microsoft Excel (Redmond, WA), and metabolite CRLBs were compared at each TE using the Wilcoxon signed‐rank test. Comparison with data from normal‐appearing prostate tissue available from the previous study^13^ used a Mann–Whitney *U*‐test.

### Metabolite Quantification

Absolute metabolite concentrations were produced from the TE = 32 msec spectra for metabolites with CRLB/amplitude <20%, by referencing metabolite amplitudes to the magnitude of the free induction decay from the matching, water‐unsuppressed scan. Water tissue concentration was assumed to be 46.1 M^15^ and *T*_1_ and *T*_2_ corrections were made using assumed *T*_1_ and *T*_2_ values from the literature (Table 3). Tumor *T*_2_ values were also calculated from TE = 32 msec and 100 msec tumor spectra judged by inspection as having good fits (ie, correct peak allocation, not fitting to noise), and assuming a monoexponential fit.

For partial‐tumor voxels the expected concentrations of citrate and spermine were calculated with Eq. (1):
(1)
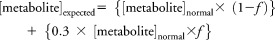



In Eq. (1), the metabolite concentration in normal tissue is from the matched prostate voxel, *f* is the tumor fraction of the voxel, and the metabolite concentration inside tumor is assumed to be 30% of that in the normal tissue.^7^

## Results

### Tumor Metabolite Depiction at TE = 32 msec vs. TE = 100 msec (Cohort 1)

**Figure 1:**
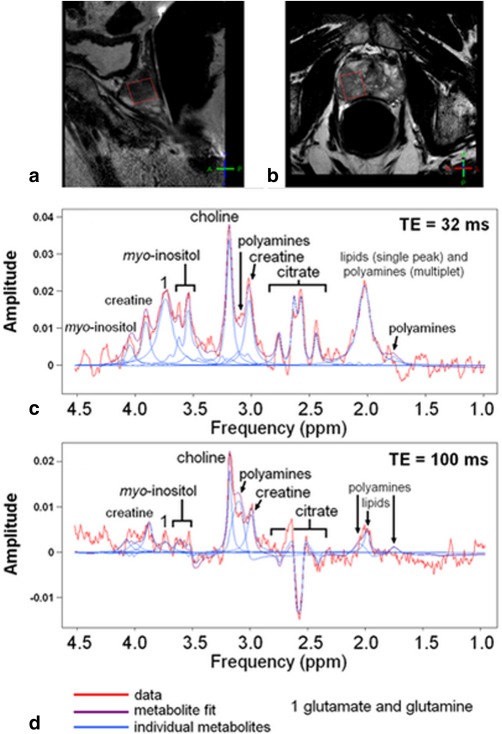
Sagittal **(a)** and transverse **(b)** *T*_2_‐weighted MR images of a prostate showing the tumor spectroscopic voxel in red. The fitted spectra from this voxel are shown at TE = 32 msec **(c)** and TE = 100 msec **(d)**, with metabolite concentrations measured at TE = 32 msec for citrate, 7.2 mM, spermine, 0.6 mM, choline, 4.6 mM, creatine, 8.0 mM, and myo‐inositol, 7.5 mM. Apodizations = 3 Hz.

Due to lipid contamination in TE = 32 msec spectra, four patients were omitted from this cohort. Unsuppressed water linewidths were less than 10 Hz, indicating good shimming. Example spectra at both TEs from a voxel consisting entirely of tumor are shown in Fig. 1. The metabolite resonances of spermine, choline, creatine, and myo‐inositol had CRLB/amplitude <20% in 19, 14, 14, and 17/22 patients, respectively at TE = 32 msec, and in 11, 19, 12, and 4/22 patients, respectively at TE = 100 msec. Citrate was depicted in all 22 TE = 32 msec spectra but, since no satisfactory model existed to fit citrate at TE = 100 msec, citrate depiction level could not be determined at this TE. A Wilcoxon signed‐rank test showed that the depictions (CRLB/amplitude) of spermine and myo‐inositol were significantly better at TE = 32 msec than at TE = 100 msec (two‐tailed *P* < 0.02; Table 1), while the choline resonance was better depicted at TE = 100 msec (two‐tailed *P* < 0.02). Metabolite *T*_2_ values in tumor were calculated from spectra judged as having good fits: spermine *T*_2_ = 96 ± 56 msec (*n* = 4) and choline *T*_2_ = 73 ± 27 msec (*n* = 5).

**Table 1:** Median (and Interquartile range) of the Cramér‐Rao Lower Bounds of Metabolites Relative to Metabolite Amplitudes From QUEST Metabolite Fitting of^1^ 22 Tumor Spectra From Cohort 1 and^2^ Normal‐Appearing Prostate Tissues

Echo time	Tissue type	Citrate	Spermine	Choline	Creatine	myo‐inositol
TE = 32 msec	Tumor	**2.3%** (1.2–3.0)	**5.1%** (3.5–6.2)	12.9% (6.3–22.5)	13.2% (7.4–29.8)	**7.1%** (5.9–10.5)
TE = 32 msec	Normal^13^	**1.3%** a **(**0.8–2.2)	**3.4%** a (2.3–6.4)	9.6% (6.8–17.1)	11.4% (8.6–16.6)	**8.6%** (6.1–20.6)
TE = 100 msec	Tumor	Unknown^b^	18.3% (6.2–100.0)	**6.3%** a (5.2–10.4)	18.1% (11.4–40.9)	77.8% (25.0–100.0)
TE = 100 msec	Normal^13^	Unknown^b^	13.5% (7.9–34.8)	13.4% (8.3–26.8)	20.0% (12.7–43.1)	78.9% (46.7–100.0)

From Basharat et al.^13^ Lower CRLBs indicate better metabolite depiction. Key: **Bold** Within that tissue type, there is significantly better depiction at that echo time.

a At that echo time, there is significantly better depiction in that tissue type.

b A satisfactory simulation of citrate could not be made for TE = 100 msec, so citrate is not quantifiable.

### Metabolite Concentrations in Voxels Containing All Tumor, Partial Tumor, and No Tumor (Cohorts 1 and 2)

**Figure 2:**
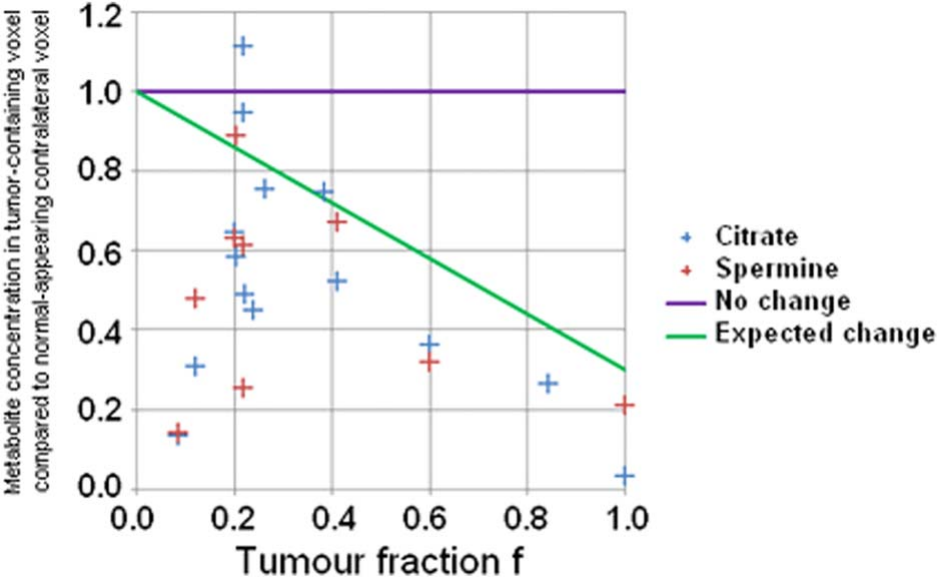
Metabolite concentration in each partial‐tumor voxel in Cohort 2 compared with the concentration in the matched, normal‐appearing prostate voxel, and expected concentration reduction using Eq. (1) (green).

In Cohort 2, due to lipid contamination, 12 patients were omitted from the analysis. Metabolite concentrations from both cohorts of patients are shown in Table 2 and metabolite *T*_2_ values from Cohort 1 in Table 3. In Cohort 2 the average tumor fraction *f* of the partial‐tumor voxels (as defined by *T*_2_W‐ and DW‐MRI in a biopsy‐positive octant) was 0.36 ± 0.27 (range 0.09–1.00). After correction for tumor fraction within each partial‐tumor voxel using Eq. (1), citrate and spermine concentrations were significantly lower than expected, by 14.3 mM (*P* = 0.02) and 4.4 mM (*P* = 0.02), respectively, compared with normal‐appearing voxels (Fig. 2). There were no significant differences in choline, creatine, or myo‐inositol concentration across these matched pairs of partial‐tumor vs. normal‐appearing voxels.

**Table 2:** Metabolite Concentrations (Mean ± SD) in mM

Morphological tissue type	Tumor		Normal‐appearing	Central gland	>75% Peripheral zone
Metabolite	Cohort 1 (full voxel)	Cohort 2 (partial voxel)	Cohort 2	Basharat et al^13^	Basharat et al^13^
Citrate	19 ± 11	25 ± 23^a^	50 ± 34	32 ± 17	64 ± 22
Spermine	6 ± 3	5 ± 3^a^	9 ± 3	7 ± 4	10 ± 4
Choline	4 ± 2	5 ± 3	4 ± 3	5 ± 4	7 ± 3
Creatine	10 ± 5	9 ± 5	9 ± 7	8 ± 7	9 ± 5
myo‐inositol	14 ± 7	13 ± 8	18 ± 5	15 ± 12	10 ± 8

**Table 3:** Literature Metabolite *T*_1_ and *T*_2_ Values Used for Concentration Correction (First Two Columns) and *T*_2_ Values in Full Tumor From Cohort 1 (Third Column) Calculated From Metabolite

Metabolite	Assumed *T*_1_	Assumed *T*_2_	Measured *T*_2_
Citrate	470^22^	170^22^	
Spermine	1025^23^	53^13^	96 ± 56 (n = 4)
Choline	1100^22^	62^13^	73 ± 27 (n = 5)
Creatine	1375^24^	209^25^	
myo‐inositol	997^24^	90^13^	

Fits judged as acceptable at TE = 32 msec and 100 msec, using a monoexponential decay model, quoted as mean ± SD (*n*).

There were correlations between citrate and spermine concentrations in the voxels consisting entirely of tumor (*n* = 22, Cohort 1, *r* = +0.66, *P* = 0.002), partial‐tumor voxels (*n* = 14, Cohort 2, *r* = +0.72, *P* = 0.02), and the normal‐appearing voxels (*n* = 14, Cohort 2, *r* = +0.61, *P* = 0.06).

### Relationships Between Metabolite Concentrations and Disease Stage and Grade

**Figure 3:**
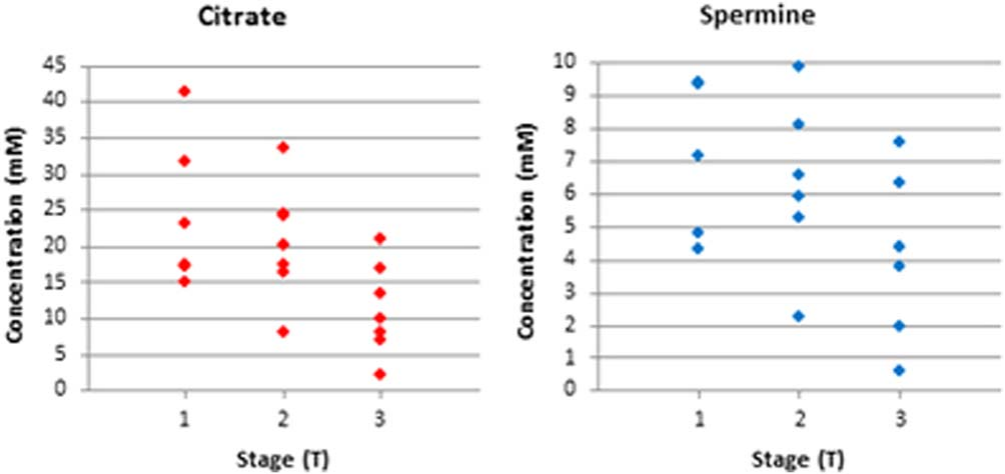
Absolute metabolite concentrations in prostate tumors of differing primary tumors stage.

In Cohort 1 (voxels consisting entirely of tumor), citrate and spermine concentrations were lower in T3 tumors (11 ± 6 mM and 4 ± 3 mM, respectively) than in both T1 (24 ± 10 mM, one‐tailed *P* = 0.01 and 7 ± 2 mM, *P* = 0.04, respectively) and T2 tumors (21 ± 8 mM, *P* = 0.03 and 6 ± 3 mM, *P* = 0.08, respectively) (Fig. 3). The concentrations of citrate in the Gleason grade 3+3, 3+4, and 4+3 tumors was 31 ± 9 mM, 12 ± 6 mM, and 18 ± 5 mM, respectively, with the concentration being significantly higher in Gleason grade 3+3 tumors than either of the other groups (*P* = 0.01). Differences between grades 3+4 and 4+3 tumors were not significant (*P* = 0.11). The concentrations of spermine in the Gleason grade 3+3, 3+4, and 4+3 tumors were 6 ± 2 mM, 4 ± 2 mM, and 7 ± 2 mM, respectively, with the concentration being significantly lower in Gleason grade 3+4 tumors than either other group (*P* = 0.03 and 0.01, Student's *t*‐test). The concentrations of the other metabolites were not significantly different between tumors of different stage.

## Discussion

This study demonstrates that there is greater depiction of the glandular metabolites citrate and spermine in prostate tumors using TE = 32 msec MRS than using TE = 100 msec MRS, due to reduced *T*_2_‐decay at TE = 32 msec (as already shown in studies of normal‐appearing prostate^13^, ^16^). The depiction of citrate and spermine signals was also worse in tumors than in other normal‐appearing prostate tissues,^13^ likely due to decreased concentrations. This reduction weakens the quality of *T*_2_ calculation in tumors (spermine had *T*_2_ = 53 ± 16 msec in normal‐appearing prostate,^13^ but a much greater standard deviation as well as a larger value in the current work, 96 ± 56 msec). Despite this reduction, it was still possible to quantify citrate and spermine individually in 100% and 86% of lipid‐free tumor voxels, respectively, at TE = 32 msec (using the criterion CRLB/amplitude <20%), compared with 50% spermine depiction at TE = 100 msec.

Choline depiction in tumors is better at TE = 100 msec than in nonmalignant tissue spectra at TE = 100 msec. This difference is likely due to the decrease in the size of the overlapping spermine resonance due to the depletion of spermine in cancer, rather than to an increase in choline concentration. Choline depiction in tumor is also better at TE = 100 msec than at TE = 32 msec. This difference is likely due to the *J*‐evolution of spermine. The 3.1 ppm multiplet of spermine at TE = 32 msec appears approximately as a triplet with relative amplitudes 0.51 (3.21 ppm), 1.00 (3.11 ppm), and 0.79 (3.02 ppm), whereas at TE = 100 msec it appears more like a broad singlet due to *J*‐evolution, with amplitudes (at the same offsets as before) of 0.06, 1.00, and −0.37. This leads to less spectral overlap of choline with spermine at TE = 100 msec.

Citrate and spermine concentrations decreased significantly from normal even in partial‐tumor voxels containing only small amounts of tumor (Fig. 2). Evidence already exists that metabolite changes may occur in the tissues adjacent to prostate tumor since, for example, prostate tumors smaller than 0.5 cm^3^ (defined by histopathology) have been assessed as being larger than 0.5 cm^3^ when using the metabolite ratio (choline + spermine + creatine)/citrate to determine the extent of metabolic abnormality.^1^, ^17^ In the present study it was observed that citrate and spermine concentrations are both systematically decreased in the tissues immediately adjacent to prostate tumor regions, defined by morphological imaging. The reduction in metabolite levels may be due to tumor field effects (although it could also be due to benign disease). Tumor field effects are a result of genetic and molecular alterations in the histologically normal tissues adjacent to tumor, which are either induced by or precursory to tumor.^18^, ^19^ If citrate and spermine concentration reductions are genuine prostate tumor field effects (precursory citrate depletion is hypothesized by Costello et al^14^) then, using MRS, small prostate tumors may be detected early and before morphological imaging techniques are capable of visualizing them.

As expected from prostate tumor extract studies,^20^ both citrate and spermine concentrations (calculated from TE = 32 msec spectra) were lower in tumors of increasing stage, although there was overlap between stages. The greatest limitation in concentration calculation, inaccurate *T*_2_ values, is mitigated in this study by using short TEs, which reduces *T*_2_ errors compared to measurement at longer TEs. Additionally, although tumor upgrading is common after initial biopsy results, citrate concentrations were also significantly higher in Gleason grade 3+3 tumors here than tumors of higher grade. Citrate and spermine reduction may therefore help indicate decreasing glandular function and higher tumor grades in vivo. Conversely, choline is an indicator of cell proliferation (choline/creatine increases in tumors of higher grade^20^) but, although there was a paucity of high‐grade tumors in this study, no significant increase was observed in choline concentrations in those few tumors of high grade, or between tumors and normal‐appearing voxels overall. These data suggest that MRS choline levels are not useful in detecting small prostate tumors in vivo, likely due to poor depiction (choline had the worst depiction of all five metabolites in tumor at TE = 32 msec).

Short TE MRSI is commonly applied in the brain but is not often used in the prostate due to the conventional inclusion of long, frequency‐selective pulses to suppress contaminating lipid signals from periprostatic fat tissues.^21^ Despite using single‐voxel MRS here, which is not subject to voxel bleeding, as in MRSI, lipids contaminated 30% of spectra. Thus, lipid contamination is a limiting aspect of this methodology and lipid suppression techniques are essential for short TE PRESS in the prostate. For example, one study used 20 very selective saturation pulses placed automatically around the prostate and achieved 86% citrate depiction at TE = 40 msec MRS (CRLB/amplitude <40%).^16^

In this study, data acquisition time was kept low, at 2–3 minutes per voxel, to ensure patient tolerance and compliance, but increasing data acquisition time if only one voxel is being studied rather than two would be acceptable.

One limitation of this study is that although the tumors were biopsy‐confirmed, the tumor regions were demarcated using MRI only (*T*_2_‐weighted imaging and diffusion) and not histologically. This may well affect the measurement by inaccurate definition of the extent of tumor within tumor‐designated voxels, as well as the partial‐voxel investigation. This is particularly problematic in low‐risk, low‐grade (Gleason grade 3+3 or 3+4) disease, as was the case with the majority of our cohort. However, there is a good evidence base for identifying prostate lesions in this way so that this should not have a large effect on the results.

With regard to the investigation of differences in metabolite content between tumors of different stage and grade, owing to the cohort of patients studied (those being studied by active surveillance), this study only included grades 3+3, 3+4, and 4+3 (with stages T1, T2, and T3). It is possible that larger differences might be observed in higher‐risk, high‐grade disease, but that remains to be determined. A further limitation is that tissue water was used as the reference for metabolite quantification. Variation in the actual water concentrations and relaxation behavior (*T*_1_ and *T*_2_ relaxation time constants) will affect the calculated metabolite concentrations. There is no particular reason for this to lead to a systematic bias, but it will have increased the spread of values obtained. In addition, the spread of values for the *T*_2_ of spermine and citrate measured in this study will include contributions both from biological variation as well as from uncertainty in the fit to the spectra.

In summary, TE = 32 msec MRS offers a way of measuring citrate and spermine resonances in over 86% of lipid‐free prostate tumor voxels, which is better than their depiction in long TE MRS, due to reduced transverse decay at short TE. Citrate and spermine levels may be useful in identifying prostate tumors as they decrease in tumors of increasing stage and grade. Choline concentration, on the other hand, was not found to be useful as a prostate tumor indicator in vivo. Citrate and spermine concentrations reduced in the normal‐appearing tissues adjacent to tumor, which may reflect a tumor field effect.
